# HMCN1 as a conserved biomarker of epithelial–mesenchymal transition: a cross-cancer analysis

**DOI:** 10.3389/fonc.2025.1730887

**Published:** 2025-12-12

**Authors:** Yuanxi Leng, Yayun Liu, Le Zhi

**Affiliations:** 1 Hand and Foot Surgery Department, Hongdu Traditional Chinese Medicine Hospital Affiliated to Jiangxi University of Chinese Medicine, Nanchang, Jiangxi, China; 2 Department of Orthopedics, Jiangxi Provincial People’s Hospital, The First Affiliated Hospital of Nanchang Medical College, Nanchang, Jiangxi, China

**Keywords:** biomarkers, EMT, hemicentin-1, osteosarcoma, pan-cancer

## Abstract

**Background:**

The extracellular matrix (ECM) critically regulates tumor progression, but the systematic role of its large constituent hemicentin-1 (*HMCN1*) across cancers remains poorly defined. Although implicated in cell invasion and migration, a comprehensive pan-cancer understanding of *HMCN1* is lacking.

**Methods:**

We performed an integrative multi-omics analysis of *HMCN1* across 33 cancer types using data from TCGA, GTEx, and CPTAC. Based on prominent functional importance in bone tumors, osteosarcoma was selected for functional validation. In vitro experiments, including knockdown and overexpression, were conducted to assess effects on migration and invasion.

**Results:**

*HMCN1* was frequently upregulated in tumors and associated with poor prognosis. Its mutations correlated with genomic instability. *HMCN1*-high tumors were enriched in inflammatory immune subtypes and exhibited a dysfunctional tumor microenvironment. Pathway analysis consistently linked *HMCN1* expression to epithelial-mesenchymal transition (EMT). Functional validation in osteosarcoma confirmed that *HMCN1* knockdown suppressed, while its overexpression enhanced, cell migration and invasion through regulation of key EMT markers.

**Conclusions:**

This study identifies *HMCN1* as a novel, conserved regulator of EMT and a candidate prognostic biomarker across cancers. The findings in osteosarcoma provide a foundational rationale for developing *HMCN1* as a potential therapeutic target.

## 1 Introduction

Cancer, a major global public health challenge arising from complex genetic and epigenetic regulatory networks, is characterized by high degrees of heterogeneity and molecular complexity, which result in an incomplete understanding of its regulatory mechanisms. In recent years, advancements in high-throughput sequencing technologies and the establishment of large-scale data-sharing platforms have positioned pan-cancer research as a key paradigm for systematically uncovering the commonalities and heterogeneity among cancers, and these technologies have led to remarkable progress (1). By integrating genomic, transcriptomic, epigenomic, and clinical data across multiple cancer types, this approach has the potential to identify shared driver mutations, signaling pathways, and potential therapeutic targets, and thereby offers new perspectives for early cancer detection, prognostic stratification, and personalized treatment. Despite these advantages, there exist certain challenges with integrating multi-omics data and interpreting their complex biological significance (2–4). Identifying key molecules with roles across multiple cancers remains a crucial goal of pan-cancer research. Such molecules can reveal previously unrecognized pan-cancer mechanisms and hold promise as broad-spectrum biomarkers or therapeutic targets (5). Further, validating their universality and effectiveness as therapeutic targets will provide a critical theoretical foundation for developing universal or adaptable targeted therapies for a variety of malignancies, as well as offer significant translational value, particularly in drug discovery and combination therapy strategies. Therefore, the identification and validation of such functional hubs or molecular markers would not only represent a crucial breakthrough in understanding the “common rules” of cancer biology but also drive the construction of a cross-cancer precision medicine framework and innovate clinical practice models. The identification of pan-cancer prognostic biomarkers represents a major focus in cancer research. Such biomarkers refer to molecular features significantly associated with patient outcomes (e.g., overall survival and progression-free survival) across different cancer types. Investigating these biomarkers aims to transcend the limitations of single-cancer-type studies, reveal shared cancer biological processes, and thereby provide a scientific basis for developing universal prognostic tools, guiding personalized treatment strategies, and improving clinical outcomes for cancer patients (6, 7).

Tumorigenesis and tumor progression are driven by genetic mutations and signaling aberrations within cancer cells, as well as by complex interactions among diverse cellular and molecular components of the tumor microenvironment (TME). Notably, the extracellular matrix (ECM) constitutes a fundamental and frequently dysregulated compartment of the TME, making it a prime source for the pan-cancer biomarkers discussed above. The extracellular matrix (ECM), a core constituent of the TME, provides structural support and actively participates in regulating cell behavior and tissue homeostasis in normal tissues. However, in tumors, its composition, structure, and mechanical properties undergo significant alterations, including aberrant expression, increased cross-linking, and enhanced degradation activity (8, 9). Collectively, these changes promote tumor cell proliferation, migration, and invasion, while also influencing immune cell infiltration and function, thereby fostering a microenvironment conducive to tumor progression (10, 11). However, given the vast complexity and heterogeneity of the ECM across different cancers, a systematic, pan-cancer approach is essential to identify which specific ECM components act as conserved regulators of malignancy. Such an analysis can pinpoint central nodes within the tumor ECM that hold promise as broad-spectrum biomarkers or therapeutic targets. Hemicentin-1 (*HMCN1*), a prominent member of the fibulin family distinguished by its large molecular weight and widespread distribution in the basement membranes of various tissues, has emerged as a key molecule in ECM research.

*HMCN1* was initially identified in *Caenorhabditis elegans* for its role in tethering adjacent basement membranes and was, subsequently, proven to play a crucial role in maintaining the integrity of the basement membrane-connective tissue boundary through studies on *HMCN1*-deficient mice (12). Further, functional studies have demonstrated that *HMCN1* directly binds to Keratin 14, and its loss was found to reduce keratin intermediate filament formation and induce subepidermal blistering in 3D skin equivalent models (13). This underscores its vital function in linking the epidermal cytoskeleton to the ECM. Emerging evidence indicates an active role for *HMCN1* in tumor progression. For example, Liu et al. reported co-upregulation of sushi repeat−containing protein, X−linked (*SRPX*) and *HMCN1* in cancer-associated fibroblasts from ovarian cancer patients. Functionally, knocking down *HMCN1* significantly suppressed Transwell invasion of cancer cells, a mechanism shown to be mediated through the activation of the *RhoA* signaling pathway in fibroblasts (14). Furthermore, in bovine leukemia virus infection models, *HMCN1* upregulation was associated with tumor cell migration (15). Together, this evidence suggests that *HMCN1* exerts multifaceted regulatory functions in the TME that extend beyond mere structural support.

However, a critical and unresolved question remains: does *HMCN1* act as a conserved, central regulator of cancer progression across diverse tumors, or merely as a context-dependent passenger? Establishing *HMCN1*’s fundamental role is of paramount scientific importance because, as a large ECM glycoprotein, it is uniquely positioned at the interface between the cellular cytoskeleton and the basement membrane. This strategic location suggests it could be a master orchestrator of tissue architecture and cell signaling. Clinically, resolving this ambiguity is crucial. If *HMCN1* is a conserved oncogenic driver, it would emerge as a novel and compelling therapeutic target, particularly for aggressive, metastatic cancers driven by EMT and immune evasion – two processes it appears to influence. Currently, few ECM components have been successfully targeted, making *HMCN1* a promising candidate for pioneering new classes of stromal therapy. Critically, it is unknown whether *HMCN1* represents a conserved oncogenic driver across cancers or a context-dependent factor, and its potential role as a master regulator of central processes like epithelial-mesenchymal transition (EMT) is poorly defined. To test the hypothesis that *HMCN1* is a pan-cancer tumor-promoting gene that promotes malignancy primarily through activating EMT, the present study conducted a comprehensive analysis of *HMCN1* characteristics across 33 cancer types by integrating data from The Cancer Genome Atlas (TCGA), Genotype-Tissue Expression (GTEx), and DepMap, as well as single-cell and spatial transcriptomic atlases. We delineated the genomic, transcriptomic, and proteomic landscapes of *HMCN1* and used the resulting data to evaluate its prognostic value and immunomodulatory functions across cancer types. Given the mesenchymal origin of osteosarcoma and its well-characterized reliance on ECM remodeling and EMT for progression, coupled with our preliminary data indicating high *HMCN1* expression and functional essentiality in bone cancer contexts, we selected this aggressive tumor type for subsequent experimental validation of *HMCN1*’s oncogenic role (16–18). Our research demonstrates its tumor-promoting properties based on published pan-cancer data as well as *in vitro* experiments in an osteosarcoma model. Importantly, *HMCN1* emerged as a novel regulator of epithelial-mesenchymal transition (EMT) in the cancer milieu. Thus, these findings establish *HMCN1* as a pan-cancer ECM-derived biomarker and a potential therapeutic target and provide a crucial foundation for future research into *HMCN1* and related ECM mechanisms.

## 2 Methods

### 2.1 Bibliometric analysis

A comprehensive literature search was conducted using the Web of Science Core Collection (WOSCC) database to systematically review studies on *HMCN1* in the field of oncology. The following Boolean search strategy was employed: [(*HMCN1* OR Hemicentin-1 OR “Hemicentin 1” OR FIBL-6 OR FIBL6 OR FBLN6 OR ARMD1) AND (cancer* OR carcinoma* OR tumor* OR tumour* OR neoplas* OR malignan* OR oncolog*)]. The search was limited to articles published between January 1, 2000, and September 1, 2025. The article types included were research articles and reviews. Comments, letters, conference abstracts, and retracted publications were excluded, and only English-language documents considered. Visualizations were generated to highlight trends in keywords and their expansions within the *HMCN1* research theme (19).

### 2.2 Pan-cancer data sources and data preprocessing

Transcriptomic data and corresponding clinical information, including overall survival (OS), progression-free interval (PFI), disease-free interval (DFI), and disease-specific survival (DSS), were retrieved from TCGA. To compare *HMCN1* expression between tumor and normal tissues, we also obtained matched normal tissue data from the GTEx project and integrated the cancer and normal datasets. Specifically, we utilized the TPM (transcripts per million) expression data from the UCSC Xena database (datasets: tcga_RSEM_gene_tpm and gtex_RSEM_gene_tpm). To ensure accuracy and account for anatomical factors, only primary tumor tissues from TCGA were paired with GTEx data. The expression data were then standardized by converting them into a unitless Z-score for each cancer type using the formula (x-μ)/σ. Outliers, defined as Z-scores less than -3 or greater than 3, were removed. A cancer type was included in the subsequent analysis only if at least three normal samples remained after outlier removal. Samples with incomplete clinical data, poor sequencing quality, or undetectable *HMCN1* expression were excluded to ensure data integrity and comparability. Using the integrated TCGA and GTEx dataset, we systematically compared differences in *HMCN1* expression between normal and malignant tissues across various cancer types.

Protein-level expression of *HMCN1* was assessed using the Human Protein Atlas platform, and three-dimensional structural information was retrieved from Protein Data Bank. Gene dependency or essentiality was assessed using CRISPR screening data from the DepMap portal. Gene essentiality was quantified using the Chronos score: a score <0 indicates that *HMCN1* knockdown is detrimental to cell survival and is suggestive of its potential role in tumorigenesis (20, 21). Pan-cancer single-cell RNA sequencing data were obtained from the TISCH database (http://tisch.compbio.cn/home/). Using the pheatmap R package, we generated a pan-cancer single-cell expression heatmap. Euclidean distance was used as the similarity metric, and Ward’s minimum variance method was employed for hierarchical clustering to investigate conserved patterns of gene expression origin. Further dimensionality reduction and visualization of the high-dimensional data were performed using the Uniform Manifold Approximation and Projection method (22).

Spatial transcriptomics data were sourced from the Sparkle database [with sample identifiers as previously described (23)]. For the spatial transcriptomics analysis, we first identified each microregion characterized by a dominant cell type and detected its gene expression profile. After Z-score normalization, the data were visualized using the pheatmap package. Next, the spatial distribution of major cell types across microregions was computed and visualized using the SpatialPlot function of the Seurat package. Finally, Spearman correlation analysis was used to determine the correlation between cellular composition and gene expression data across all the detected spots with the linkET package, and the results were visualized to illustrate correlation patterns.

### 2.3 Analysis of the diagnostic and prognostic potential of *HMCN1*

The diagnostic utility of *HMCN1* was evaluated by constructing receiver operating characteristic (ROC) curves using the pROC package, and its prognostic value was assessed through Kaplan-Meier survival analysis (survival and survminer packages) and univariate/multivariate Cox regression analyses (forestplot package). The restricted cubic spline (RCS) method was employed to analyze potential non-linear relationships between variables. This statistical approach flexibly captures complex relationships between a continuous independent variable (*HMCN1* expression in this case) and a dependent variable (survival time in this case) by fitting cubic polynomials at multiple knots and connecting them smoothly. In this study, RCS was incorporated into the Cox proportional hazards model to test for non-linear associations between *HMCN1* expression and the hazard ratio (HR). The significance of any non-linear effect was assessed via hypothesis testing to enhance model accuracy and interpretability (24).

Based on data extracted from the TIDE database, the association of *HMCN1* expression levels with response to immunotherapy and prognosis was analyzed to evaluate its potential as a predictor of immunotherapy response. The association of *HMCN1* with immunotherapy response was assessed using data from the TIDE web platform. This analysis integrated transcriptomic and clinical data from a pan-cancer cohort (comprising non-small cell lung cancer, melanoma, and bladder cancer) who received anti-PD-1 or anti-CTLA-4 therapy. Patient response was defined based on the objective response rate according to RECIST criteria, as pre-computed by the TIDE framework.

### 2.4 Genomic mutation analysis

Single nucleotide variation (SNV) data for 33 cancer types were collected from TCGA. The analyzed mutation types were Missense_Mutation, Silent, 5’ Flank, 3’ UTR, RNA, In_Frame_Del, Nonsense_Mutation, Splice_Site, Intron, 5’ UTR, In_Frame_Ins, Frame_Shift_Del, Nonstop_Mutation, 3’ Flank, Frame_Shift_Ins, and Translation_Start_Site. The SNV mutation frequency (expressed as a percentage) for each gene coding region was calculated using the following formula: (number of mutated samples/total number of samples for that cancer type) (25). Somatic copy number alterations for *HMCN1* were analyzed using GISTIC 2.0 (26) on data from the TCGA pan-cancer cohort, with thresholds set as previously described (|G-score| > 0.1) (27). Data on structural variations (SVs) were obtained from the pre-computed TCGA which aggregates calls from established algorithms. Mutation spectrum oncoplots and lollipop plots were generated utilizing the cBioPortal database and the maftools R package, respectively, with protein domain information sourced from the PFAM database (https://www.ebi.ac.uk/interpro/). To further investigate the correlation between different gene sets and *HMCN1* expression across the pan-cancer datasets, Spearman correlation analysis was employed. The resulting correlation coefficients and their corresponding statistical significance were visualized using radar plots (28, 29).

### 2.5 Analysis of the role of *HMCN1* in the TME

To systematically investigate the role of *HMCN1* in the TME and its clinical implications, we integrated multi-dimensional analytical approaches. Samples were stratified into high- and low-*HMCN1* groups based on the median expression level. The association between these groups and the six established immune subtypes (C1–C6) was then assessed using a Chi-square test. Subsequently, to more precisely delineate the relationship between *HMCN1* expression gradients and immune features, patients were further stratified into four quartiles (Q1–Q4, where Q1 represents the highest 25% and Q4, the lowest 25%) based on *HMCN1* expression levels. The average values of various immune scoring metrics were calculated for each quartile (with missing values ignored), and the expression patterns of immune features were visualized using the pheatmap package to comprehensively display the continuous trends between *HMCN1* expression levels and the immune status of the TME (25). By leveraging the TIMER2.0 database, Spearman correlations between *HMCN1* and different immune-infiltrating cells were computed using multiple algorithms, and the results were intuitively presented in a heatmap.

### 2.6 Identification of potential *HMCN1*-targeting anti-tumor drugs

To explore potential therapeutic strategies targeting *HMCN1*-mediated tumor promotion, we performed a Connectivity Map (CMAP) analysis. By comparing patients with high versus low *HMCN1* expression, we identified the 150 most significantly upregulated and 150 most significantly downregulated genes to construct an *HMCN1*-related gene signature. This signature was then matched against the perturbation profiles of 1,288 compounds in the CMAP database. Similarity scores were calculated using the XSum (eXtreme Sum) algorithm, and compounds with lower scores were considered potential candidates for reversing the *HMCN1*-mediated pro-tumor phenotype (30).

### 2.7 Enrichment analysis and pathway activity assessment

This study utilized the CancerSEA database, a resource that systematically integrates single-cell transcriptomic data reflecting 14 distinct functional states of cancer cells. The comprehensive score for each functional state was calculated using the z-score method from the Gene Set Variation Analysis (GSVA) R package. After standardization, this score was defined as the respective gene set activity index. Subsequently, the Pearson correlation between individual gene expression and these gene set activity scores was analyzed (31). Protein expression data were obtained from The Cancer Proteome Atlas. Patients were stratified into high and low *HMCN1* protein expression groups based on the median expression level. Differences in pathway activity scores between these two groups were compared using the Wilcoxon test. Additionally, Gene Set Enrichment Analysis (GSEA) was performed using the clusterProfiler R package to further investigate the underlying biological mechanisms (32).

### 2.8 Cell culture and real-time quantitative PCR analysis of the mRNA expression of *HMCN1*

Human osteosarcoma (Saos-2, MG63, HOS, U-2OS, 143B) and osteoblastic (HOB) cell lines were obtained from the Cell Bank, Chinese Academy of Sciences (all certified) and cultured in Dulbecco modified Eagle medium supplemented with 10% fetal bovine serum and 1% penicillin-streptomycin at 37°C in a 5% CO_2_ atmosphere.

The mRNA expression level of *HMCN1* in cells was determined by real-time quantitative PCR (RT-qPCR). Total RNA was extracted from cells using the TRIzol reagent (Vazyme Biotech, Nanjing, China, Cat# R401-01). cDNA was synthesized from 1 µg of total RNA using the HiScript III RT SuperMix for qPCR (+gDNA wiper) kit (Vazyme Biotech, Cat# R323-01) according to the manufacturer’s instructions. RT-qPCR amplification was performed on a QuantStudio 5 Real-Time PCR System (Applied Biosystems, USA) using the ChamQ Universal SYBR qPCR Master Mix (Vazyme Biotech, Cat# Q711-02). The reaction conditions were as follows: initial denaturation at 95°C for 30 s, followed by 40 cycles of 95°C for 10 s and 60°C for 30 s. *GAPDH* served as the internal reference gene. The relative gene expression levels were calculated and analyzed using the 2^–ΔΔCt method. All primers utilized in this study were synthesized by Wuhan Servicebio Technology Co. Ltd. The primer sequences were as follows: *HMCN1* (NM_031935.3) forward: ACTGATGCTCGGTCCAAAGATTA, reverse: CTTGACCAGAACTTGTAGAGGCA (amplicon: 163 bp); *GAPDH* (NM_002046.7) forward: GGAAGCTTGTCATCAATGGAAATC, reverse: TGATGACCCTTTTGGCTCCC (amplicon: 168 bp).

### 2.9 Western blot assay of *HMCN1* and EMT-related proteins

Total proteins were extracted from a panel of osteosarcoma cell lines (Saos-2, MG63, HOS, U-2OS, 143B) and a human osteoblast cell line (HOB) as a control. Cells were washed with cold PBS and lysed on ice using RIPA Lysis Buffer (Beyotime Biotechnology, Shanghai, China, Cat# P0013B) supplemented with 1 mM phenylmethylsulfonyl fluoride (PMSF, Beyotime, Cat# ST506) and a protease inhibitor cocktail (MedChemExpress, China, Cat# HY-K0010). The lysates were centrifuged at 12,000 × g for 15 min at 4°C, and the supernatants were collected. To manipulate *HMCN1* expression, cells were transfected with three distinct *HMCN1*-specific siRNAs (si-*HMCN1*-1, si-*HMCN1*-2, si-*HMCN1*-3) designed and synthesized by GWuhan Servicebio Technology Co. Ltd (China) or an *HMCN1* overexpression construct (OE-*HMCN1*), using non-targeting siRNA and an empty vector as negative controls. The siRNA sequences were as follows: si-*HMCN1*-1: 5’-GCAUCAAGAUUACCGUUUATT-3’; si-*HMCN1*-2: 5’-CCUGUACAAGAGGACCAUUTT-3’; si-*HMCN1*-3: 5’-GGAGAAGUUCUACAACAAATT-3’. The protein concentration of all lysates was quantified using a bicinchoninic acid (BCA) assay kit (Beyotime, Cat# P0012). Subsequently, equal amounts of protein (10-30 µg per lane) were separated by 8-12% sodium dodecyl sulfate-polyacrylamide gel electrophoresis and electrophoretically transferred onto polyvinylidene difluoride membranes (Millipore, USA) under a constant current of 300 mA. The membranes were blocked with 5% non-fat milk for 2 h at room temperature and then incubated overnight at 4°C with specific primary antibodies diluted in TBST containing 5% bovine serum albumin. The primary antibodies and their dilutions were as follows: anti-*HMCN1* (1:1000, Thermo Fisher Scientific, Cat# PA5-149932), anti-E-cadherin (1:2000, CST, #3195), anti-N-cadherin (1:1500, Proteintech, 22018-1-AP), anti-Vimentin (1:1000, CST, #5741), anti-Snail (1:1000, CST, #3879), and anti-*GAPDH* (1:5000, Proteintech, 60004-1-Ig), as previously described (33). After thorough washing, the membranes were incubated with appropriate horseradish peroxide-conjugated secondary antibodies (1:5000, Abbkine, China). Protein bands were visualized using an enhanced chemiluminescence (ECL) detection kit (New Cell & Molecular Biotech, Suzhou, China, Cat# P10300) and the band intensity was quantified with the ImageJ software (National Institutes of Health, USA). *GAPDH* was used as an internal loading control for normalization.

### 2.10 Wound healing and transwell invasion and migration assays

For the wound healing assay, 5 × 10^5^ cells were seeded per well in 6-well plates. Once the cells reached 100% confluence, uniform scratches were created across pre-marked lines using a sterile 200 µL pipette tip. The plates were washed with phosphate-buffered saline to remove detached cells and then replaced with serum-free medium to minimize cell proliferation. Images of the same fields were captured at 0 h and 24 h using an inverted microscope (Olympus, Japan). The wound closure area was quantified using ImageJ software. For the Transwell invasion assay, Transwell chambers (Corning, USA, Cat# 3422) with 8-µm pores were pre-coated with Matrigel (Corning, Cat# 356234) diluted 1:8 in serum-free medium, and solidified at 37°C for 4 hours. Serum-free medium containing 1 × 10^5^ cells was added to the upper chamber, while the lower chamber was filled with medium supplemented with 20% fetal bovine serum as a chemoattractant. Following 24 h of incubation, non-invading cells on the upper surface were carefully removed with a cotton swab. The cells that had invaded through the Matrigel were fixed with 4% paraformaldehyde for 30 minutes, stained with 0.1% crystal violet for 20 minutes, and subsequently quantified by counting cells in five random fields under the microscope. The migration assay was performed similarly but without Matrigel coating. All assays were performed in triplicate and repeated three times independently.

### 2.11 Statistical analysis

All statistical analyses were conducted using the R 4.4.1 software and GraphPad Prism 8.4.0. Group comparisons were performed as follows: Student’s *t*-test for parametric data, Wilcoxon test for non-parametric data, Chi-square or Fisher’s exact test for categorical variables, and one-way ANOVA or Kruskal-Wallis test for comparison of multiple groups. Correlations were assessed using Pearson’s (normal distribution) or Spearman’s (non-normal distribution) methods. Survival was analyzed using Cox proportional hazards regression and log-rank tests. In the meta-analysis, heterogeneity was quantified with Cochran’s Q and I². I^2^ >50% was considered to indicate significant heterogeneity (p < 0.05), in which case a random-effects model was applied. In case of I^2^ <50%, a fixed-effects model was used. Statistical significance was defined as p < 0.05.

## 3 Results

### 3.1 Multi-dimensional characterization of *HMCN1* localization and its pan-cancer expression patterns at the gene, protein, cellular, and tissue levels

**Figure 1:**
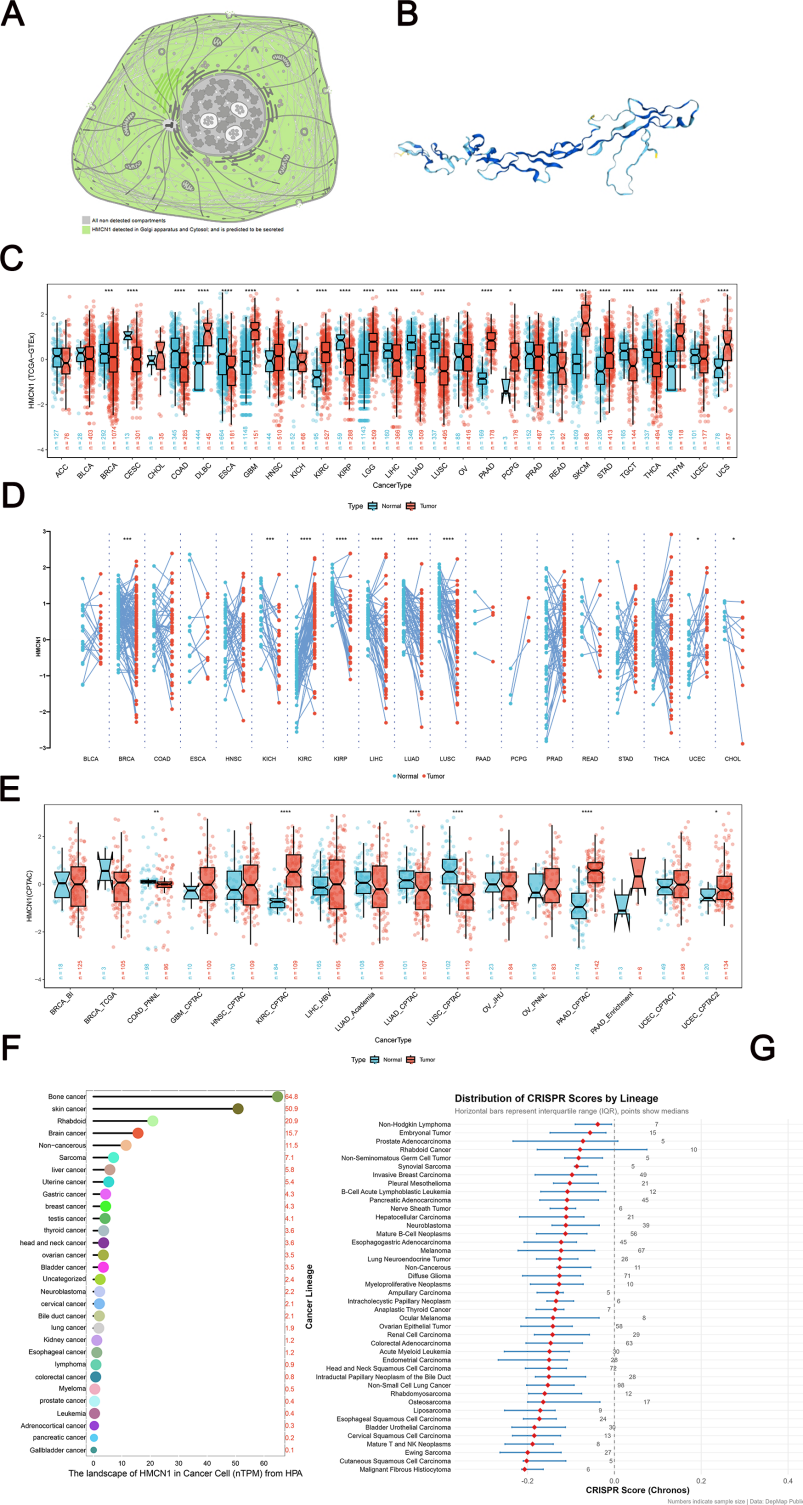
Expression and localization of HMCN1 across cancers. **(A)** Subcellular localization of HMCN1 as annotated in the UniProt database. **(B)** Three-dimensional structure of HMCN1 from the PDB database. **(C)** HMCN1 mRNA expression levels in tumor tissues from The Cancer Genome Atlas (TCGA) compared to normal tissues from the Genotype-Tissue Expression (GTEx) project. Statistical significance was determined using the unpaired two-tailed Student’s t-test. **(D)** Paired comparison of HMCN1 mRNA levels between tumor tissues and their matched adjacent normal tissues from the TCGA pan-cancer dataset. Statistical significance was determined using the paired two-tailed Student’s t-test. **(E)** Differential HMCN1 protein expression levels between tumor and normal tissues across various cancer types in the Clinical Proteomic Tumor Analysis Consortium (CPTAC) dataset. Statistical significance was determined using the unpaired two-tailed Student’s t-test. **(F)** The landscape of HMCN1 expression (nTPM: normalized transcripts per million) in cancer cell lines from the Human Protein Atlas (HPA) database. **(G)** Gene essentiality of HMCN1 across cell lines from various organs, as quantified by Chronos loss-of-function scores from the DepMap portal. Data are presented as mean ± SD. ^*^p < 0.05, ^**^p < 0.01, ^***^p < 0.001, ^****^p < 0.0001.

Bibliometric analysis indicated that *HMCN1* oncological research remains nascent, even though it has recently gained traction. Moreover, the published literature largely focuses on prognosis, mutation, and therapy (**Supplementary Figures 1A, B**). Accurate identification of the subcellular localization of *HMCN1* is crucial for a deeper understanding of its biological significance, as gene function is dependent on its subcellular localization (34). Localization analysis based on the UniProt database indicated that HMCN1 is primarily distributed in the Golgi apparatus and cytoplasm, which is consistent with its biosynthesis and secretion pathway as a large extracellular matrix glycoprotein. Its functional deposition into the extracellular matrix and basement membrane is well-established, as demonstrated in models of skin and tendon junctions (12). Concurrently, we obtained three-dimensional structural information of this protein from Protein Data Bank (**Figures 1A, B**), and this was used as a structural basis for further functional investigations, which is critical to note that this model is not experimentally verified and covers only a portion of the full-length protein. Structural information should be regarded as a putative visual guide rather than a definitive structural basis.

Next, the mRNA expression level of *HMCN1* was compared between various tumor tissues and corresponding normal tissues using data from the TCGA and GTEx databases. The results showed that *HMCN1* mRNA expression was significantly elevated in cancers such as glioblastoma multiforme (GBM), skin cutaneous melanoma (SKCM), and kidney renal clear cell carcinoma (KIRC) (**Figure 1C**). Furthermore, paired analysis using data from the TCGA pan-cancer dataset consistently demonstrated that *HMCN1* mRNA expression was markedly upregulated in most tumor tissues compared to adjacent normal tissues (**Figure 1D**). To determine whether this genetic dysregulation also manifests at the protein level, we integrated and analyzed pan-cancer proteomics data from CPTAC (https://pdc.cancer.gov/pdc/browse). The results revealed that although *HMCN1* protein expression showed an upward trend in most cancer types, this dysregulation was statistically significant only in a few cases (**Figure 1E**). To further clarify its expression characteristics at the protein level, we performed supplementary validation using the Human Protein Atlas dataset. The results suggested that *HMCN1* expression was highest in bone cancer cell lines (**Figure 1F**). This explains the lack of significant results in the CPTAC dataset. Analysis using Chronos loss-of-function scores indicated that *HMCN1* plays important functional roles in various malignancies, including osteosarcoma (**Figure 1G**). These findings indicate its potential involvement in carcinogenesis as a tumor-associated gene.

Multi-omics technologies, particularly the application of single-cell sequencing, have significantly enhanced the precision of detecting gene expression patterns (35). Therefore, we applied this technology to determine the expression of *HMCN1* in specific cell types by analyzing pan-cancer single-cell RNA sequencing data from the TISCH database. The results showed that *HMCN1* is primarily expressed in fibroblasts, endothelial cells, and a subset of tumor cells (**Supplementary Figure 1C**). To further validate this expression pattern and confirm it spatially, we integrated and analyzed pan-cancer spatial transcriptomics data. The results confirmed significant *HMCN1* expression in the aforementioned cell types (**Supplementary Figure 1D**).

Building on the above results, we investigated the spatial distribution heterogeneity of *HMCN1* within the TME. Using spatial transcriptomics data from GBM, SKCM, and KIRC, we categorized tissue regions into three types based on the proportion of malignant cells within each microregion (spot): regions with a malignant cell proportion of 1 were defined as malignant regions; those with a proportion of 0, as normal regions; and the rest, as mixed malignant regions (23). The SpatialPlot function of the Seurat package was used to visualize the major cell types and *HMCN1* expression distribution across these microregions (36). The results demonstrated that *HMCN1* expression levels were significantly higher in malignant regions than in normal and mixed regions. Further, Spearman correlation analysis of the correlation between cellular composition and *HMCN1* expression levels across all microregions, performed using the linkET package, revealed a significant positive correlation between *HMCN1* expression and malignant cell content in tumor cell-enriched areas (**Supplementary Figure 2**). In summary, *HMCN1* exhibits an overexpression pattern in various cancer tissues, and its expression is spatially concentrated in malignant areas in a way that is highly consistent with the distribution of tumor cells. This suggests that *HMCN1* holds potential as a clinical diagnostic biomarker, a possibility that was explored in the following experiments.

### 3.2 *HMCN1* as a valuable prognostic and diagnostic biomarker across cancer types

**Figure 2:**
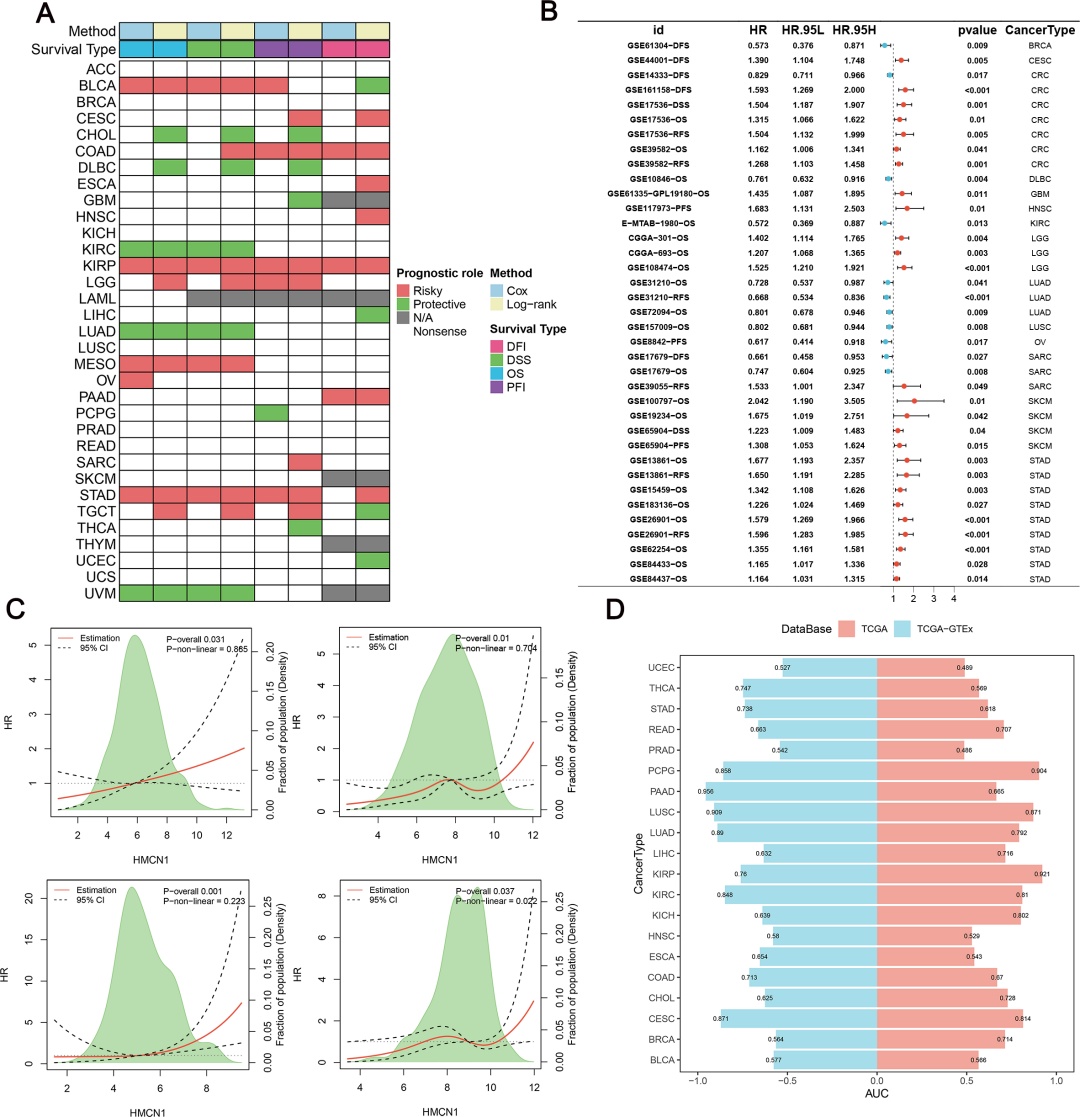
Diagnostic and prognostic value of HMCN1 in various cancers. **(A)** Heatmap of hazard ratios (HRs) from univariate Cox regression analysis, illustrating the association between HMCN1 mRNA expression and overall survival (OS), disease-specific survival (DSS), progression-free interval (PFI), and disease-free interval **(DFI)** across cancer types in the TCGA cohort. Red indicates HR > 1 (risk factor), blue indicates HR < 1 (protective factor). **(B)** Correlation of HMCN1 mRNA levels with patient overall survival in independent Gene Expression Omnibus (GEO) datasets. **(C)** Restricted cubic spline (RCS) analysis evaluating the continuous relationship between HMCN1 expression levels and the hazard ratio for overall survival (OS) in bladder urothelial carcinoma (BLCA), stomach adenocarcinoma (STAD), kidney renal papillary cell carcinoma (KIRP), and pancreatic adenocarcinoma (PAAD). The solid red line indicates the hazard ratio (HR), and the dashed black lines represent the 95% confidence interval (CI). P-overall and P-nonlinear values from the Cox proportional hazards model are shown. **(D)** Diagnostic performance of HMCN1 expression in distinguishing tumor from normal tissues, as shown by area under the curve (AUC) values of receiver operating characteristic (ROC) curves for various cancer types.

Using data from the TCGA database, we first assessed the association between *HMCN1* expression and tumor status using univariate logistic regression analysis. The results showed that in multiple cancer types, including GBM, SKCM, and KIRC, the odds ratios were all greater than 1. This indicates that elevated *HMCN1* expression is significantly associated with the tumor state, reflecting its differential expression between malignant and normal tissues. (**Table 1**). Subsequently, OS, DSS, PFI, and DFI were analyzed using Cox proportional hazards regression models and the log-rank test. OS analysis indicated that high *HMCN1* expression was significantly associated with shortened OS in patients with BLCA, KIRP, MESO, OV, and stomach adenocarcinoma (STAD). Furthermore, *HMCN1* was also a significant risk factor for worsened DFI in these cancers. With regard to PFI, high *HMCN1* expression was an independent risk factor for COAD and KIRP (**Figure 2A**). To further validate the prognostic value of *HMCN1*, we performed validation in multiple independent external datasets. The results consistently demonstrated that HMCN1 is a significant unfavorable prognostic factor in various cancers. However, in KIRC, despite upregulated *HMCN1* expression across multiple datasets, it exhibited a protective prognostic effect (**Figure 2B**). This paradoxical association might reflect tissue-specific biological functions of *HMCN1*, potentially related to its role in maintaining structural integrity in renal tissues, though the precise mechanisms warrant further investigation.

**Table 1:** Univariate logistic regression.

Variable	OR_confidence_interval	*P*-value
ACC	0.813(0.585-1.132)	2.202372e-01
BLCA	0.828(0.540-1.269)	3.869203e-01
LGG	8.542(6.788-10.748)	9.197628e-75^*^
BRCA	0.714(0.613-0.833)	1.765374e-05
CESC	0.228(0.107-0.488)	1.390167e-04
CHOL	1.219(0.541-2.750)	6.327674e-01
COAD	0.397(0.323-0.487)	8.177123e-19
DLBC	5.059(3.106-8.242)	7.425406e-11^*^
ESCA	0.559(0.468-0.668)	1.523590e-10
GBM	22.952(14.720-35.790)	1.821131e-43^*^
HNSC	1.251(0.907-1.725)	1.731479e-01
KICH	0.663(0.429-1.026)	6.486308e-02
KIRC	3.879(2.913-5.167)	1.828058e-20^*^
KIRP	0.338(0.226-0.505)	1.268227e-07
LIHC	0.559(0.443-0.705)	8.902403e-07
LUAD	0.079(0.056-0.113)	5.782136e-45
LUSC	0.057(0.038-0.084)	1.827580e-45
OV	0.931(0.726-1.194)	5.743806e-01
PAAD	27.323(14.462-51.619)	2.184343e-24^*^
PCPG	2.773(0.974-7.894)	5.597392e-02
PRAD	0.864(0.704-1.060)	1.609725e-01
READ	0.517(0.398-0.670)	6.611666e-07
SKCM	10.395(6.958-15.530)	2.943458e-30^*^
STAD	2.654(2.140-3.293)	7.058655e-19^*^
TGCT	0.423(0.311-0.575)	4.002846e-08
THYM	5.375(3.846-7.512)	6.942791e-23^*^
THCA	0.303(0.242-0.379)	2.181346e-25
UCS	3.171(1.966-5.114)	2.213370e-06^*^
UCEC	0.813(0.619-1.069)	1.384209e-01

Association between HMCN1 expression and tumor status across various cancers using univariate logistic regression. This analysis was performed using transcriptomic and clinical data from The Cancer Genome Atlas (TCGA) to evaluate the potential of HMCN1 expression as a diagnostic biomarker. An Odds Ratio (OR) > 1 indicates that higher HMCN1 expression is associated with an increased probability of the sample being tumor tissue.

* indicates a P-value less than 0.05, meaning the result is significant at the 0.05 level.

The potential non-linear relationship between *HMCN1* expression levels and OS was thoroughly investigated with the flexible RCS method (24). The results showed that in cancers such as BLCA, STAD, KIRP, and PAAD, the effect of *HMCN1* expression on survival risk followed a linear trend. The red solid line in the figure indicates that the HR increases linearly with rising *HMCN1* expression, with a statistically significant overall association observed (*P*-overall < 0.05) (**Figure 2C**). The result of the non-linearity test was not significant (*P*-non-linear > 0.05); thus, there was no apparent non-linear effect of this gene in the aforementioned cancers. This finding suggests a stable and consistent exposure-response relationship between *HMCN1* expression levels and patient prognosis in the case of these cancers. Its impact pattern demonstrates good interpretability and predictability and supports its use as a reliable linear prognostic indicator for subsequent research and clinical risk modeling (**Figure 2C**).

Previous studies have indicated that in STAD, the phosphatidylinositol-4,5-bisphosphate 3-kinase catalytic subunit alpha (*PIK3CA*) and *HMCN1* co-mutation significantly reduced patient mortality risk. When this combination further co-occurred with low-density lipoprotein receptor-related protein 1B (*LRP1B*) or AHNAK nucleoprotein 2 (*AHNAK2*) mutations, distinct survival curves were observed. The survival results implied that *HMCN1* mutation might exert a protective effect by influencing signaling pathways or microenvironment stability in the context of synergistic mutations. However, the role of *HMCN1* alone remained unclear in the previous study (37). Therefore, this study used a comprehensive supplementary analysis to investigate further the role of *HMCN1* in STAD. Analysis based on the GSE13861, GSE15459, and GSE26901 datasets revealed that high *HMCN1* expression was significantly associated with higher mortality (**Supplementary Figure 3A**). A meta-analysis further confirmed that high *HMCN1* expression was significantly correlated with poorer overall survival in patients with STAD (pooled HR = 1.23, 95% confidence interval [CI] = 1.16–1.30, *p* < 0.01; **Supplementary Figure 3B**). Multivariate Cox regression analysis showed that after adjusting for multiple clinical variables, high *HMCN1* expression remained significantly associated with adverse prognosis (HR > 1, *p* < 0.05); thus, it could serve as an independent prognostic factor in STAD (**Supplementary Figure 3C**). Finally, ROC curve analysis confirmed that *HMCN1* has high accuracy in distinguishing tumor from normal tissue and is applicable to most cancer types (**Figure 2D**). Overall, these findings demonstrate that *HMCN1* holds diagnostic and prognostic value across different cancer types.

### 3.3 Genomic alteration analysis of *HMCN1*

**Figure 3:**
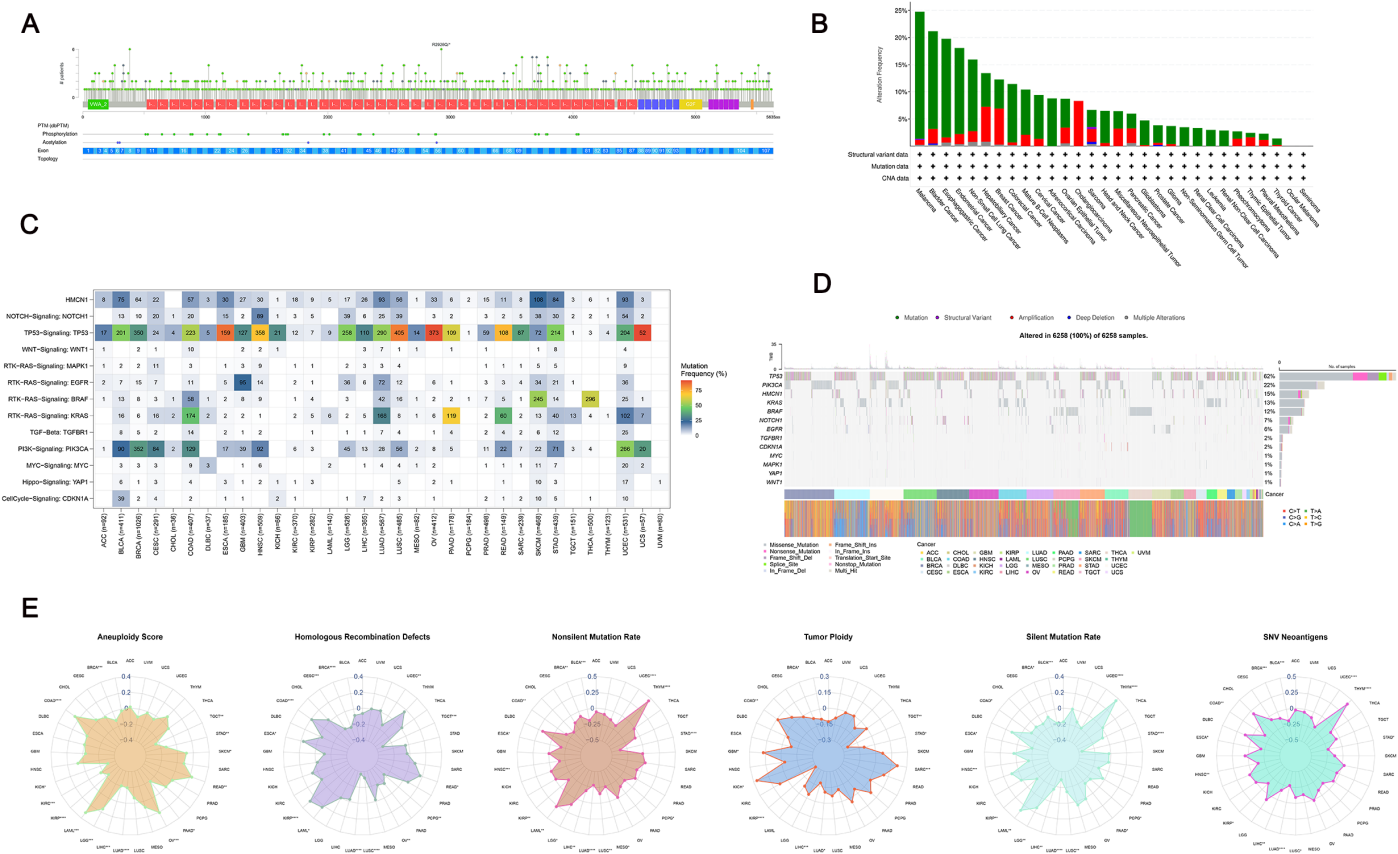
Genomic alteration profiles of HMCN1. **(A)** Panoramic view of HMCN1 mutation sites across different cancers. **(B)** Stacked bar plot representing the frequency of HMCN1 mutations in pan-cancer analyses. **(C)** Heatmap displaying the co-occurrence of HMCN1 mutations with key oncogenic signaling pathways. **(D)** Mutation distribution profile (oncoplot) of HMCN1 across cancer types. **(E)** Radar plot illustrating the Spearman correlation coefficients between HMCN1 expression and multiple genomic instability scores (e.g., aneuploidy score, fraction altered, homologous recombination deficiency) across the TCGA pan-cancer dataset.

We first mapped the mutation spectrum of *HMCN1* across pan-cancer datasets by color coding the circles corresponding to each mutation type in the plot (**Figure 3A**). Further analysis based on the cBioPortal database revealed differential mutation frequencies of *HMCN1* across various cancer types. Specifically, *HMCN1* gene amplification was primarily observed in cholangiocarcinoma, while deep deletions and structural variants were more prevalent in sarcoma (**Figure 3B**). Analysis of SNV data from the TCGA database indicated that *HMCN1* exhibits a moderate mutation frequency in several cancers, with the frequency being particularly high in SKCM (**Figure 3C**). A mutation landscape generated by the maftools package revealed that missense mutations constitute the predominant mutation type for *HMCN1* (**Figure 3D**). Furthermore, Spearman correlation analysis revealed a significant positive association between *HMCN1* expression levels and various genomic instability scores across multiple cancers. It is important to note that this observed association could be confounded by underlying factors such as tumor purity and proliferation rate. Nonetheless, this pan-cancer pattern suggests a potential link between *HMCN1* expression and a genomic context characterized by higher chromosomal instability. These associations were intuitively presented via a radar chart, which highlighted the potential value of *HMCN1* as a therapeutic target (**Figure 3E**).

### 3.4 Role of *HMCN1* in the tumor immune microenvironment and therapeutic implications

**Figure 4:**
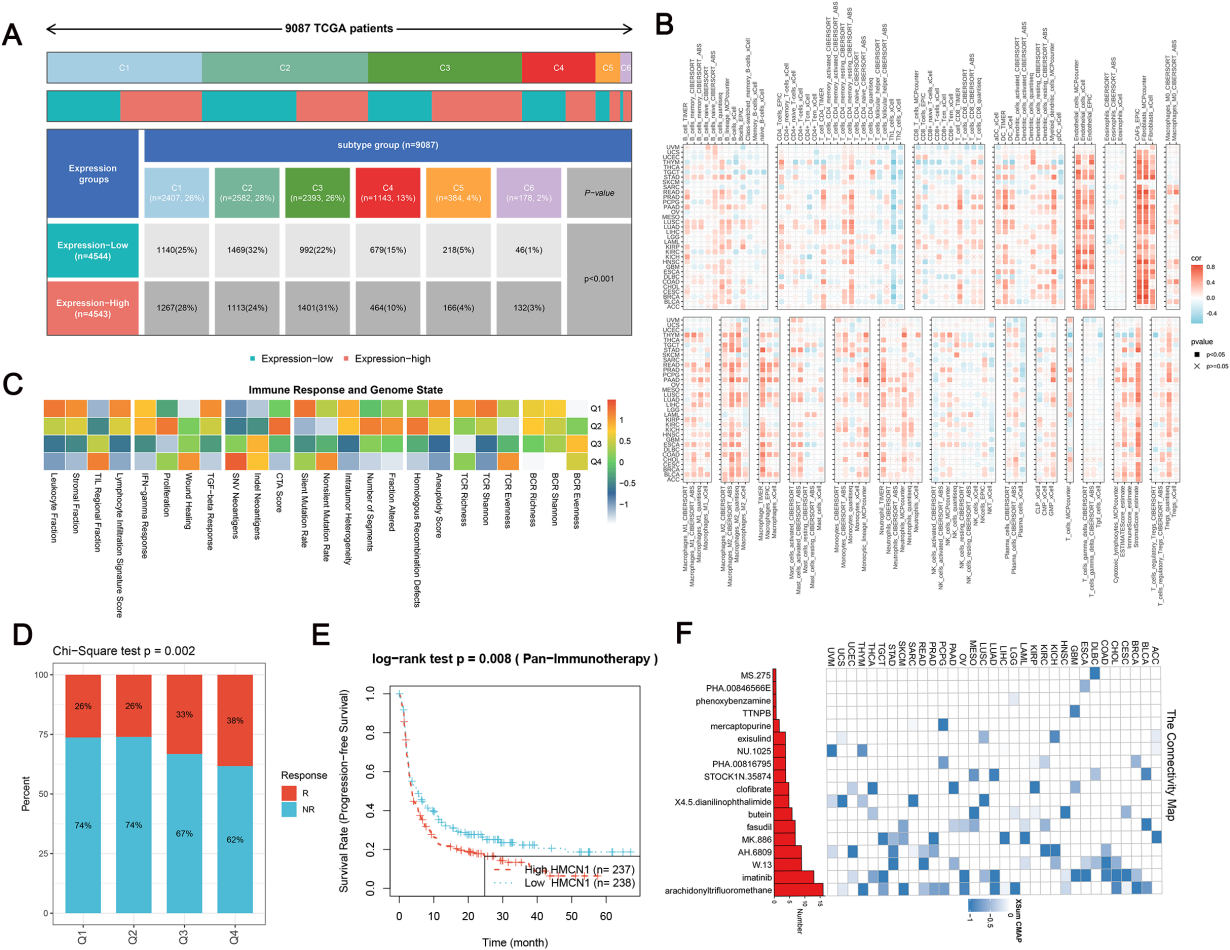
HMCN1 in immune regulation, therapy resistance, and drug targeting. **(A)** Stacked bar chart showing the distribution of six immune subtypes (C1-C6) across tumors stratified by high and low HMCN1 expression. Association was assessed by Chi-square test. **(B)** Positive associations between HMCN1 levels and infiltration of multiple immune cell types in various cancers. **(C)** Integrated analysis linking HMCN1 expression with genomic traits and immune infiltration patterns. **(D, E)** High HMCN1 expression is associated with adverse outcomes in the absence of immunotherapy and reduced treatment response, indicating a possible role in therapy resistance. **(F)** Identification of *arachidonyltrifluoromethane* as a potential therapeutic agent targeting HMCN1.

Analysis of the relationship between *HMCN1* expression levels and the distribution of immune subtypes revealed that in tumors with high *HMCN1* expression, the proportion of the C3 subtype (an inflammatory subtype, characterized by high expression of Th17- and Th1-related genes and typically associated with weaker tumor proliferation inhibition) was significantly increased, whereas the proportion of the C5 subtype (an immunologically quiet subtype) was lower (**Figure 4A**) (25). This distribution pattern, combined with the poor prognosis of *HMCN1*-high patients, led us to hypothesize that this immune-infiltrated microenvironment is functionally impaired. To test this, we assessed the relationship between *HMCN1* and markers of T-cell exhaustion. We found that HMCN1 expression was significantly positively correlated with the levels of key immune checkpoint and exhaustion genes, including PDCD1 (PD-1) and CTLA4 (**Supplementary Figure 4**). This indicates that the HMCN1-high TME is not one of effective anti-tumor immunity, but rather a state of T-cell dysfunction and exhaustion, which facilitates immune evasion and explains the associated poor outcomes.

Previous studies have emphasized the crucial role of the TME in tumor progression and treatment response (38). Based on this, we employed multiple algorithms to further evaluate the correlation between *HMCN1* and the abundance of various cell types in the tumor microenvironment. The results showed that *HMCN1* had significant positive correlations with the infiltration levels of multiple immune cell types as well as stromal cells, such as endothelial cells, across the pan-cancer datasets (**Figure 4B**). By integrating this information with the aforementioned immune subtype characteristics, we hypothesize that this inflammatory microenvironment might represent a “dysfunctional” or “ineffective immune activation” state. That is, the substantial immune cell infiltration does not lead to effective killing of tumor cells but, instead, indirectly promotes tumor progression through sustained pro-inflammatory signaling. To systematically validate this hypothesis, we analyzed the association between *HMCN1*, genomic status, and immune infiltration patterns based on immunogenicity scores, DNA damage scores, and *HMCN1* expression quartiles, and the results further supported the above observations (**Figure 4C**).

Our analysis revealed that patients who did not respond to immunotherapy exhibited high *HMCN1* expression and were associated with a worse prognosis (**Figures 4D, E**). This observation suggests that high *HMCN1* expression might be linked to immunotherapy resistance. Conversely, patients with low *HMCN1* expression showed the highest rate of effective response to immunotherapy. This finding implies that targeted inhibition of *HMCN1* could represent a novel sensitization strategy. Thus, *HMCN1* could be regarded as a potential combination target for immunotherapy and offers a new direction for pan-cancer immunotherapy strategies. Following this, we conducted predictive screening for *HMCN1*-specific drugs with tumor sensitivity. Pan-cancer analysis indicated that arachidonyltrifluoromethane (also known as Arachidonyl trifluoromethyl ketone, AACOCF3; CAS# 149301-79-1) had the highest sensitivity across multiple cancers and might have potential as a targeted intervention drug (**Figure 4F**). This compound is a recognized inhibitor of cytosolic phospholipase A2 (cPLA2), with a known mechanism involving the blockade of arachidonic acid release. It is critical to clarify that this identification stems from a computational analysis of the Connectivity Map (CMAP) database, and the precise connectivity score and false discovery rate (FDR) for this specific prediction are unavailable. Most importantly, this prediction has not been experimentally validated and thus remains a hypothesis-generating finding for future research.

### 3.5 Multi-dimensional validation of *HMCN1* association with pan-cancer progression and the EMT pathway

**Figure 5:**
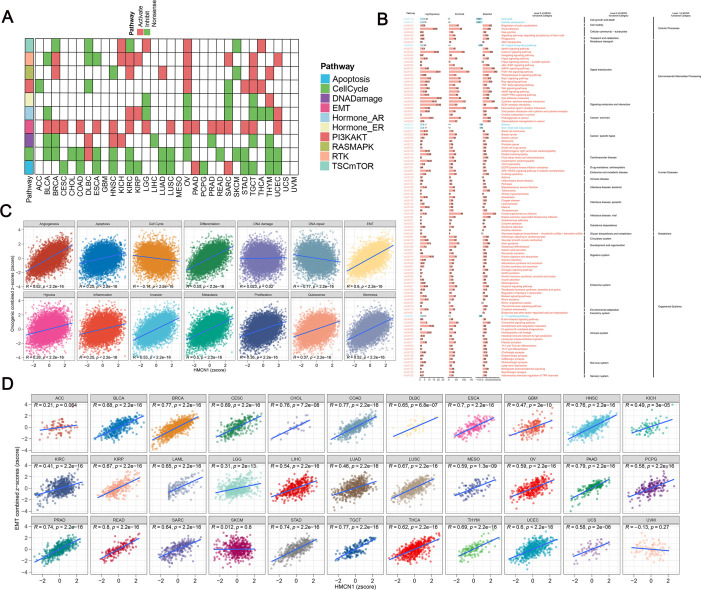
HMCN1 as a candidate conserved regulator of epithelial–mesenchymal transition (EMT). **(A)** Pathway activity analysis reveals elevated EMT scores in tumors with high HMCN1 expression. **(B)** Enrichment of multiple oncogenic signaling pathways in HMCN1-high expressing groups. **(C)** Correlation analysis from the CancerSEA database showing HMCN1 association with functional states such as angiogenesis, EMT, invasion, and stemness. **(D)** Pan-cancer analysis confirms a significant positive correlation (assessed by Spearman correlation) between HMCN1 expression and EMT activity across 33 cancer types in the TCGA dataset.

Analysis based on protein expression data from The Cancer Proteome Atlas and pathway activity scores revealed that in the vast majority of cancer types, the high *HMCN1* expression groups showed significant enrichment in EMT-related pathways, while cell cycle pathway activity was relatively lower (**Figure 5A**). This suggests that high *HMCN1* expression is associated with malignant tumor progression and is linked to the EMT process rather than directly by driving cell proliferation during cancer advancement. Further enrichment analysis indicated that multiple oncogenic signaling pathways were significantly enriched in the high *HMCN1* expression group, whereas cell cycle-related pathways were more active in the low expression group, consistent with the above findings (**Figure 5B**). To delve deeper into its functional states, we analyzed the activity of 14 cancer functional states using the CancerSEA database. Detection via GSVA and the z-score-based algorithm confirmed that *HMCN1* expression was positively correlated with pro-tumor processes such as EMT, angiogenesis, invasion, and metastasis, while simultaneously confirming its negative correlation with cell cycle activity (**Figure 5C**).

Previous studies have shown that the ECM can drive EMT through various mechanisms, thereby promoting tumor progression and metastasis, and indicate that ECM remodeling is a key driver of the EMT process in the TME. To investigate the role of *HMCN1* in this process, we further analyzed the correlation between its expression levels and EMT scores across 33 cancers. The results demonstrated that, except in the case of uveal melanoma, *HMCN1* showed a positive correlation with EMT scores in all the other cancer types examined. This suggests that *HMCN1* expression is a highly conserved indicator of EMT activity across cancer types. (**Figure 5D**).

### 3.6 Role of *HMCN1* as an tumor-promoting gene in osteosarcoma by driving malignant progression through EMT

**Figure 6:**
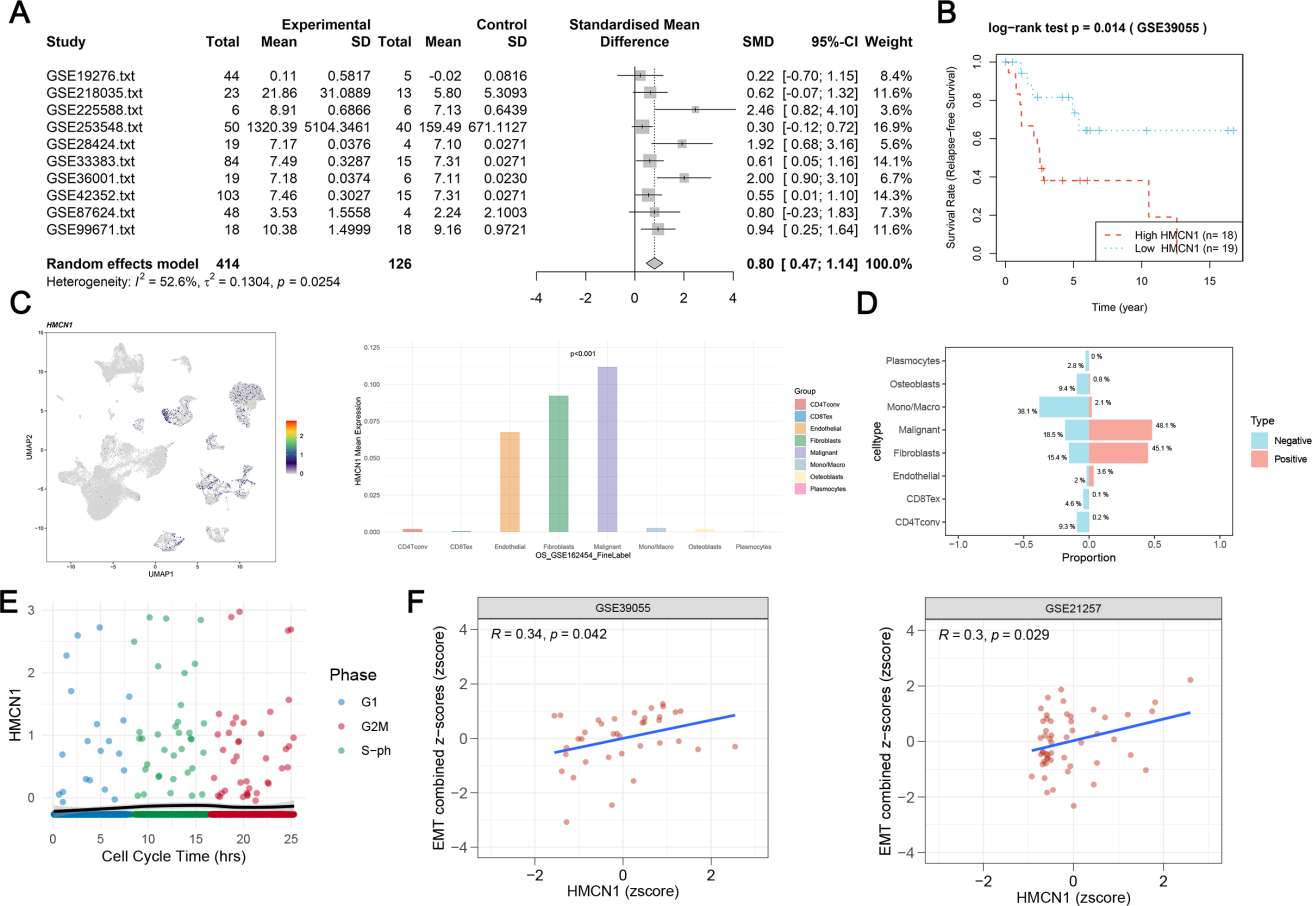
HMCN1 in osteosarcoma: prognostic and functional insights. **(A)** Meta-analysis demonstrates significant upregulation of HMCN1 mRNA in osteosarcoma tissues compared to non-osteosarcoma controls across multiple gene expression datasets. **(B)** Elevated HMCN1 expression correlates with reduced patient survival. **(C, D)** Single-cell RNA sequencing reveals highest HMCN1 expression in malignant cells, with enrichment in HMCN1-positive populations. **(E)** HMCN1 expression is not influenced by cell cycle phase. **(F)** Positive correlation between HMCN1 levels and EMT functional scores in osteosarcoma, assessed by Spearman correlation analysis in two independent GEO datasets.

No studies have yet investigated *HMCN1* expression in osteosarcoma or its impact on patient prognosis. Therefore, we focused our analysis on its mechanism of action in this tumor. We integrated mRNA expression profiles from 10 data platforms comprising a total of 540 cases, including 414 osteosarcoma and 126 non-osteosarcoma cases, and the results showed that *HMCN1* mRNA expression was significantly higher in the osteosarcoma group than in the non-osteo-sarcoma group (standardized mean difference = 0.80, 95% CI: 0.47–1.14; *p* = 0.024) (**Figure 6A**). Further, prognostic analysis indicated that high *HMCN1* expression was significantly associated with poor prognosis in patients with osteosarcoma (**Figure 6B**).

Next, we investigated the cellular heterogeneity of osteosarcoma and distribution of *HMCN1* expression across cell types through single-cell transcriptome sequencing analysis. The results revealed that *HMCN1* expression was the highest in malignant osteosarcoma cells (**Figure 6C**). We further identified all cell subpopulations and classified them into *HMCN1*-positive and *HMCN1*-negative groups based on *HMCN1* expression levels. We then calculated the proportion of each cell subpopulation within these two groups to identify the primary cell types contributing to *HMCN1* expression. Malignant cells constituted a significantly higher proportion of *HMCN1*-positive cell populations (48.1%) than *HMCN1*-negative cell populations (18.5%); this implies that malignant cells are probably the primary source of *HMCN1* expression (**Figure 6D**). These results provide important cellular-level evidence for further investigating the role of *HMCN1* in osteosarcoma development and progression.

We note that the RPPA platform employed by TCPA utilizes a predefined antibody panel, whose coverage for specific EMT-related phospho-proteins may be limited. Therefore, to more directly and definitively test the relationship between HMCN1 and EMT, we turned to functional validation. Our preliminary findings suggest that *HMCN1* might drive tumor progression primarily by promoting the EMT process rather than directly regulating cell proliferation. To validate this mechanism in the context of osteosarcoma, we utilized the GSE146773 dataset. We sorted 1,152 U2OS FUCCI cells via fluorescence-activated cell sorting and performed single-cell RNA sequencing using the SMART-seq2 technology to simultaneously obtain gene expression profiles and cell cycle phase information for individual cells (39). The results showed that there was no significant variation in *HMCN1* expression levels across different cell cycle phases (**Figure 6E**), indicating that its expression is not regulated by the cell cycle. Furthermore, in both the GSE39055 and GSE21257 datasets, *HMCN1* expression showed significant positive correlations with EMT functional state scores (**Figure 6F**). This strongly supporting a potential role for *HMCN1* in the EMT process during osteosarcoma progression (as observed from the pan-cancer data analysis).

### 3.7 *In vitro* evidence of *HMCN1*-mediated malignant behavior via regulation of EMT in osteosarcoma

**Figure 7:**
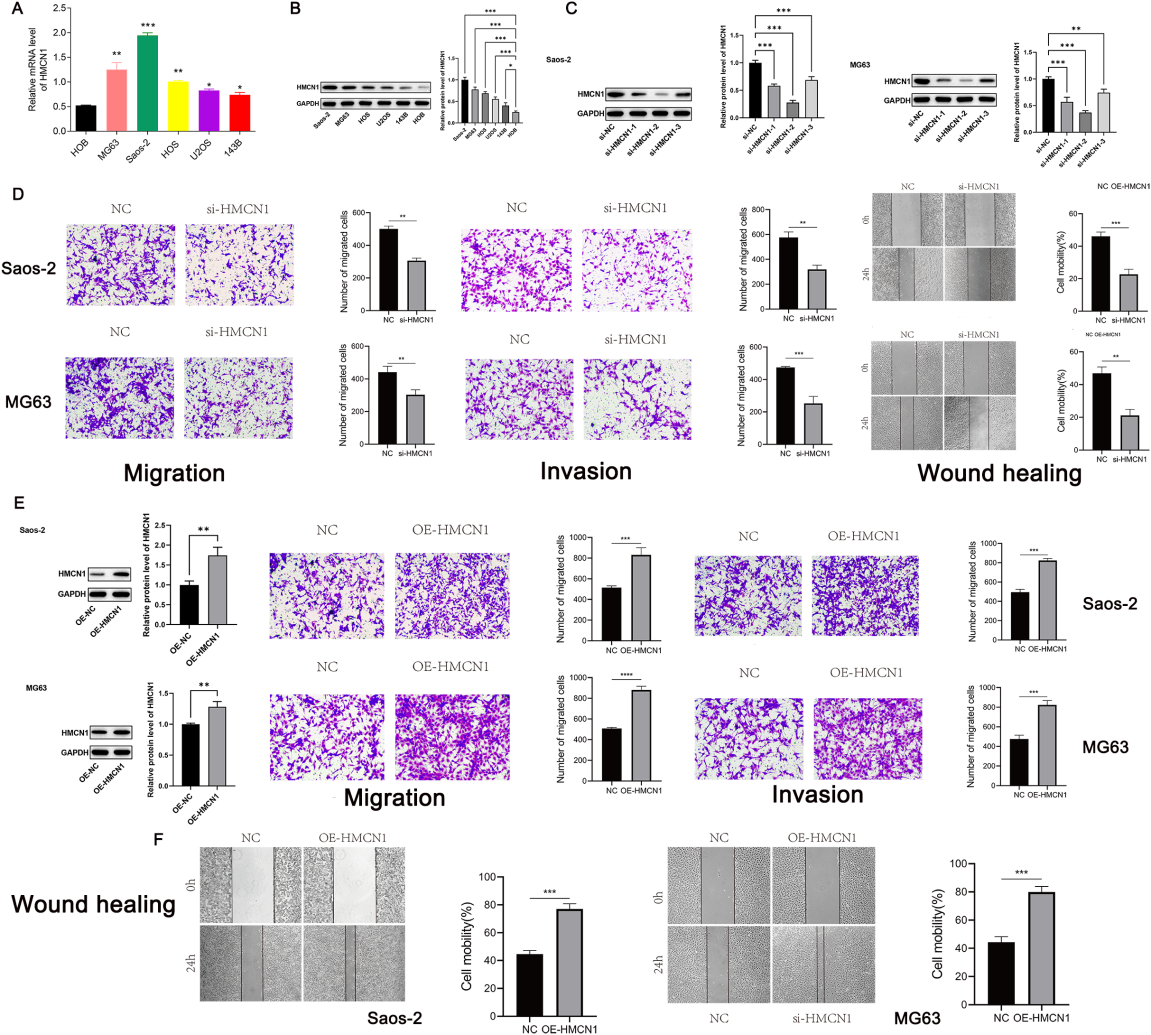
HMCN1 promotes migration and invasion of osteosarcoma cells *in vitro*. **(A, B)** HMCN1 expression is upregulated in osteosarcoma cell lines at the **(A)** mRNA and **(B)** protein levels. **(A)** Data are presented as mean ± SD (n=3). Statistical significance was determined using one-way ANOVA. **(C)** Western blot confirming efficient HMCN1 knockdown in Saos-2 and MG63. **(D)** Functional assays reveal that HMCN1 knockdown significantly inhibited the migration and invasion capabilities of MG63 and Saos-2 cells. Data are presented as mean ± SD (n=3). **(E, F)** Western blot confirming efficient HMCN1 overexpression in Saos-2 and MG63 and Functional assays reveal that HMCN1 overexpression significantly enhances the migration and invasion capabilities of MG63 and Saos-2 cells. Functional assays reveal that HMCN1 overexpression significantly enhances the migration and invasion capabilities of MG63 and Saos-2 cells. Data are presented as mean ± SD (n=3). Statistical significance was determined using the unpaired two-tailed Student’s t-test.

**Figure 8:**
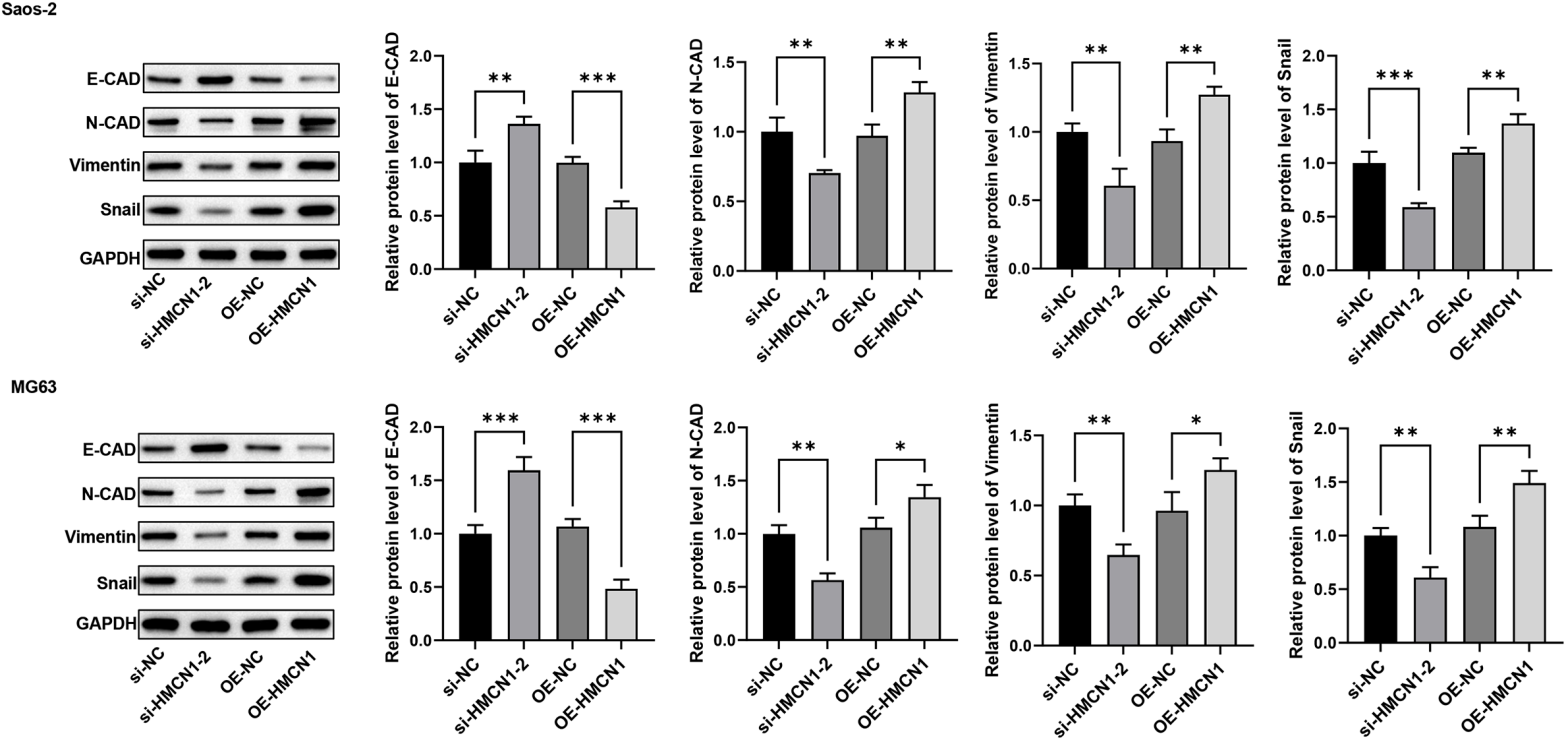
HMCN1 modulates the expression of key EMT markers. Protein levels of E-cadherin, N-cadherin, Vimentin, and Snail in osteosarcoma cells after HMCN1 knockdown or overexpression, as determined by western blot. The blots are representative of three independent experiments. * indicates a P-value less than 0.05, meaning the result is significant at the 0.05 level. **: indicates a P-value less than 0.01, meaning the result is significant at the 0.01 level. ***: indicates a P-value less than 0.001, meaning the result is highly significant at the 0.001 level.

To functionally validate the role of *HMCN1* in driving malignancy via regulation of EMT in osteosarcoma, we conducted a series of *in vitro* experiments. We first confirmed that *HMCN1* was significantly overexpressed at both the mRNA and protein levels in osteosarcoma cell lines using RT-qPCR and western blot analysis: MG63 and Saos-2 cells showed the most prominent upregulation of *HMCN1* in comparison to cells (**Figures 7A, B**). Based on this finding, we constructed three distinct *HMCN1*-targeting siRNAs and one overexpression vector. si-*HMCN1–*2 demonstrated the most potent knockdown efficiency at the *HMCN1* protein level and was, therefore, selected for all subsequent functional experiments (**Figure 7C**). Wound healing and Transwell assay results indicated that knocking down *HMCN1* significantly inhibited the migration and invasion capabilities of osteosarcoma cells, whereas its overexpression produced the opposite, promotive effect (**Figures 7D–F**). To further explore the underlying mechanism, we examined the impact of *HMCN1* on key EMT markers and found that *HMCN1* overexpression significantly upregulated the expression of N-cadherin, vimentin, and Snail, while downregulating E-cadherin. Conversely, *HMCN1* knockdown had the opposite effect. These functional gains and losses, coupled with the consistent shifts in EMT marker expression, demonstrate that *HMCN1* promotes osteosarcoma cell invasion and migration by regulating the EMT process (**Figure 8**).

## 4 Discussion

Our pan-cancer analysis establishes *HMCN1* as a critical oncogenic ECM component that is frequently upregulated across cancers, with its high expression serving as a potential prognostic biomarker that is associated with poorer survival in a linear manner. Genomic analyses revealed that *HMCN1* mutations are predominantly missense and positively correlate with genomic instability. Furthermore, its high expression was associated with immune cell infiltration and a tumor microenvironment compatible with a dysfunctional phenotype. The apparent discrepancy between HMCN1’s ECM function and its Golgi/cytoplasmic localization is reconciled by recognizing that the latter reflects its biosynthetic processing as a secreted protein, while its mature, functional form operates in the ECM. Thus, it may promote tumor development by modulating an immune context compatible with an immunosuppressive niche, a finding that aligns with and substantiates the broader role of the ECM. Mechanistically, the ECM component *HMCN1* expression is strongly associated with the pro-metastatic EMT pathway across cancers. This finding is grounded in the established role of the ECM in driving EMT across cancers, such as pancreatic (40) and breast malignancies (41, 42). Our pan-cancer analysis demonstrated a significant association between *HMCN1* expression, EMT pathway activity, and higher EMT scores. We specifically extended this finding to osteosarcoma, where the ECM’s role in promoting an EMT-like, invasive phenotype is recognized (16–18). We experimentally confirmed that *HMCN1* promotes osteosarcoma malignancy by regulating EMT. Collectively, our work elevates *HMCN1* from a mere ECM constituent to a key regulatory node and potential therapeutic target within the central ECM–EMT axis of cancer progression.

Another interesting finding that emerged from this study is that high *HMCN1* expression may not directly play a role in regulating cell cycle activity. This phenomenon may be related to the overall dysfunction of the ECM in the TME, with potential mechanisms including dysregulated ECM component expression, excessive protease degradation, abnormal cell surface receptors, and altered ECM physical properties (8, 43). In this context, as one of many components of the ECM, the potential independent regulatory function of *HMCN1* may be masked or compensated for by broader ECM dysregulation, thereby explaining the lack of significant association with cell cycle control. Transwell migration, invasion, and wound healing assays demonstrated that *HMCN1* overexpression significantly enhanced malignant behaviors in osteosarcoma. This suggests its tumor-promoting effect is probably achieved indirectly by inducing EMT, rather than through directly accelerating cell cycle progression. Numerous studies have confirmed that the EMT process is closely associated with maintaining cancer stem cell properties, and stem-like cells typically possess enhanced self-renewal capacity and proliferative potential (44). Therefore, *HMCN1* may activate the EMT program, enabling cells to acquire an invasive phenotype that subsequently manifests as a proliferative advantage overall-a mechanism that was experimentally validated in our study. Finally, drug sensitivity analysis indicated that *arachidonyltrifluoromethane* shows promise as a potential therapeutic agent targeting *HMCN1*. This compound has demonstrated anti-tumor potential in various cancer models, possibly through specifically modulating the immune microenvironment or inhibiting relevant signaling pathways to impede tumor progression. (45–47). However, it is crucial to note that our computational prediction identifies this compound based on its functional antagonism of the *HMCN1* gene signature; the precise molecular mechanism remains to be elucidated. A key question for future research is whether arachidonyltrifluoromethane exerts its effects by directly binding to the *HMCN1* protein itself or indirectly through upstream regulators or co-components within the ECM-EMT axis. These findings suggest its potential value for treating tumors with high *HMCN1* expression, although this requires additional experimental validation.

## 5 Limitations

While our study has the enormous advantage of a pan-cancer analysis of data from multiple databases and provides valuable insights into the broad role of *HMCN1*, there are several limitations that need to be acknowledged. First, the analytical approaches used may be inherently limited by batch effects or systematic discrepancies across platforms. Furthermore, our proteomic analysis is constrained by the coverage of the TCPA antibody panel. Second, although our study confirms the significant role of *HMCN1* in osteosarcoma progression, its precise molecular mechanisms require further elucidation. For instance, the dysregulation mechanisms of *HMCN1* under the influence of upstream regulators and its interaction patterns with other core molecules within the EMT pathway remain to be clarified. Finally, our functional findings lack *in vivo* validation, and future translational research should build upon our findings by exploring two key avenues. First, the anti-tumor efficacy of the identified candidate, arachidonyltrifluoromethane, requires functional validation *in vitro* to directly assess its ability to inhibit *HMCN1*-mediated oncogenic phenotypes. Second, expanding on this, the therapeutic potential of combining such *HMCN1*-targeting strategies with existing conventional treatments for osteosarcoma should be thoroughly investigated.

## 6 Conclusion

Through a systematic pan-cancer analysis, this study establishes for the first time that elevated *HMCN1* expression is a conserved biomarker positively associated with EMT across cancer types. Further, its role as an independent prognostic biomarker and oncogenic driver was validated in multiple cancers. In osteosarcoma in particular, *HMCN1* was highly expressed and found to promote malignant phenotypes, and its function as an EMT-associated tumor-promoting gene was validated. Moreover, high *HMCN1* expression was correlated with an immunosuppressive microenvironment and resistance to immunotherapy; thus, it may hold potential as a combination target for immunotherapy. Integrating data on the expression levels of this protein into clinical decision-making could provide a basis for precise treatment strategies and potentially improve patient outcomes.

## Data Availability

The datasets presented in this study can be found in online repositories. The names of the repository/repositories and accession number(s) can be found in the article/**Supplementary Material**.

## Supplementary material

Supplementary Figure 1 **(A, B)** Bibliometric analysis of HMCN1 in oncology, showing the rising publication trend and the current research focus on prognosis, mutation, and therapy. **(C, D)** Single-cell RNA sequencing and spatial transcriptomics reveal the expression pattern of HMCN1.

Supplementary Figure 2 HMCN1 expression is spatially correlated with malignant cells in the tumor microenvironment. Spatial transcriptomics analysis of GBM, SKCM, and KIRC shows that HMCN1 expression is significantly higher in malignant regions than in normal or mixed regions. Spearman correlation analysis reveals a significant positive correlation between HMCN1 expression and malignant cell content across all microregions.

Supplementary Figure 3 High expression of HMCN1 is an independent prognostic factor for poor survival in STAD. **(A)** Analysis of the GSE13861, GSE15459, and GSE26901 datasets reveals that high HMCN1 expression is significantly associated with higher mortality. **(B)** A meta-analysis confirms that high HMCN1 expression is significantly correlated with poorer overall survival in STAD patients (pooled HR = 1.23, 95% CI = 1.16–1.30, p < 0.01). **(C)** Multivariate Cox regression analysis demonstrates that high HMCN1 expression remains an independent prognostic factor for adverse outcomes after adjusting for clinical variables.

Supplementary Figure 4 The heatmap shows the correlation analysis between HMCN1 and PDCD1 and CTLA4 at the pan-cancer level.
