# Supplementary figures and images for: HMCN1 as a conserved biomarker of epithelial–mesenchymal transition: a cross-cancer analysis
Source: Front Oncol. 2025 Dec 12;15:1730887. doi: 10.3389/fonc.2025.1730887 (PMC12740748; doi:10.3389/fonc.2025.1730887)

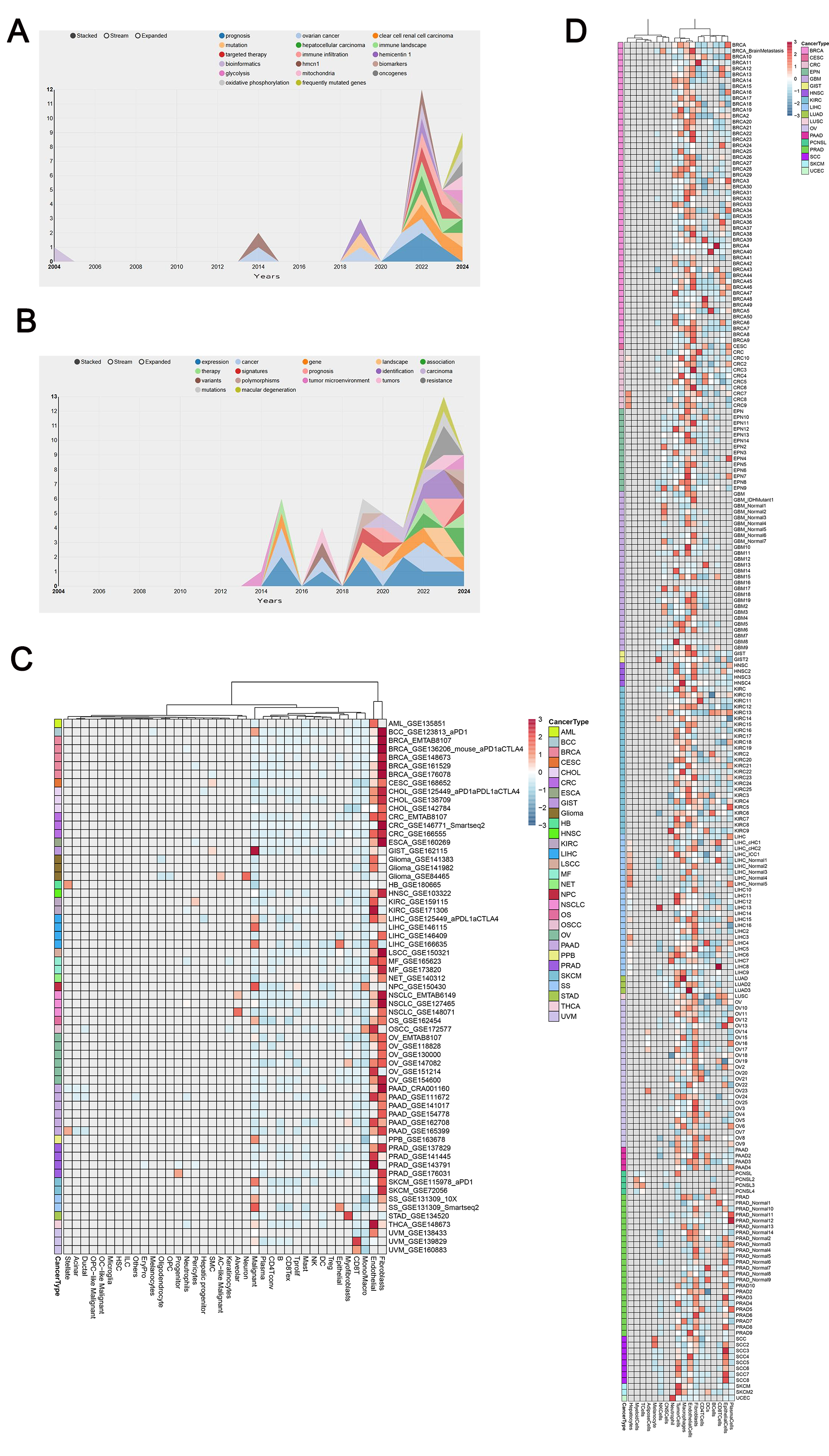

Supplement: Supplementary Figure 1 — (A, B) Bibliometric analysis of HMCN1 in oncology, showing the rising publication trend and the current research focus on prognosis, mutation, and therapy. (C, D) Single-cell RNA sequencing and spatial transcriptomics reveal the expression pattern of HMCN1. [file DataSheet1.zip › Supplementary files/Supplementary Figure 1.tif]

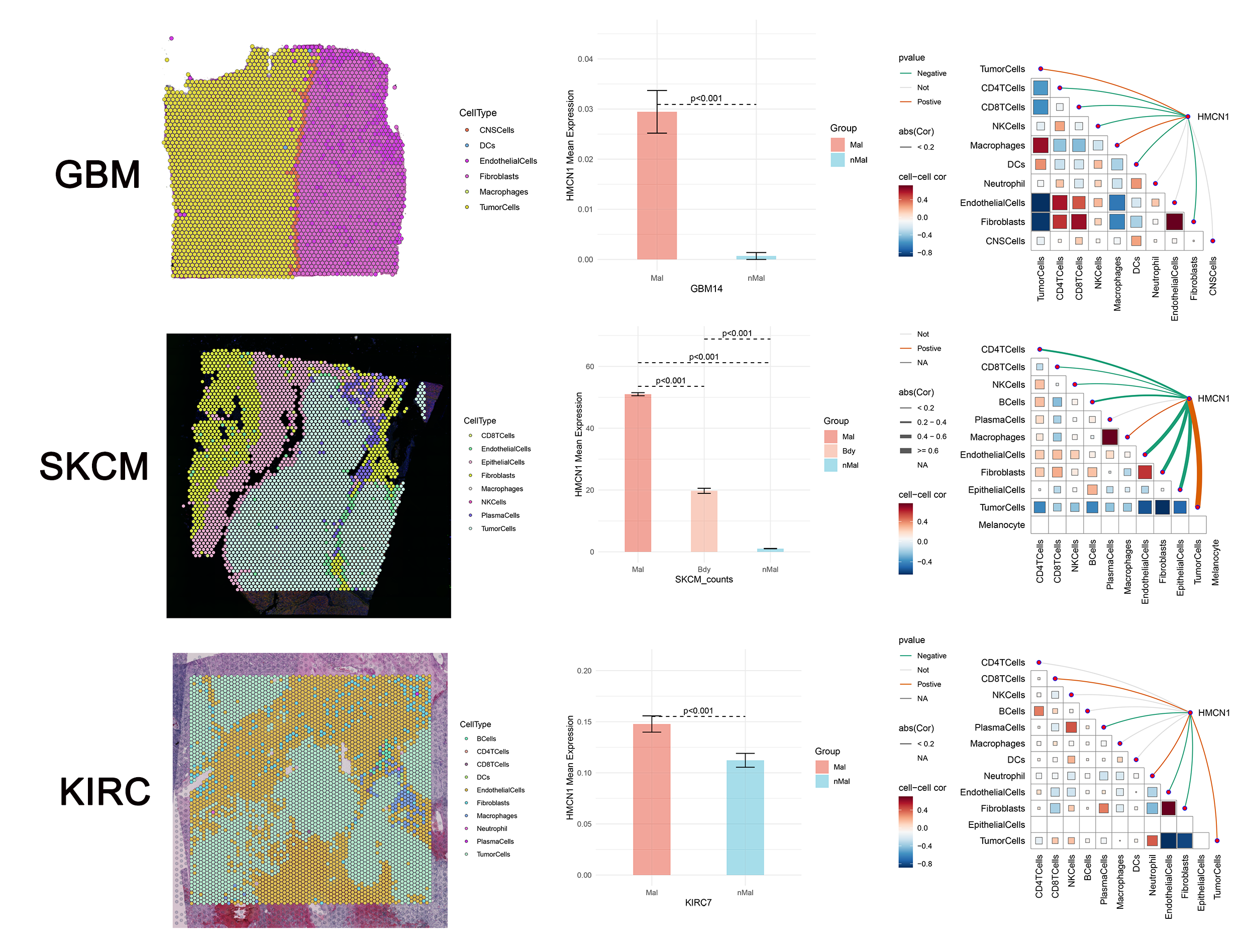

Supplement: Supplementary Figure 1 — (A, B) Bibliometric analysis of HMCN1 in oncology, showing the rising publication trend and the current research focus on prognosis, mutation, and therapy. (C, D) Single-cell RNA sequencing and spatial transcriptomics reveal the expression pattern of HMCN1. [file DataSheet1.zip › Supplementary files/Supplementary Figure 2.tif]

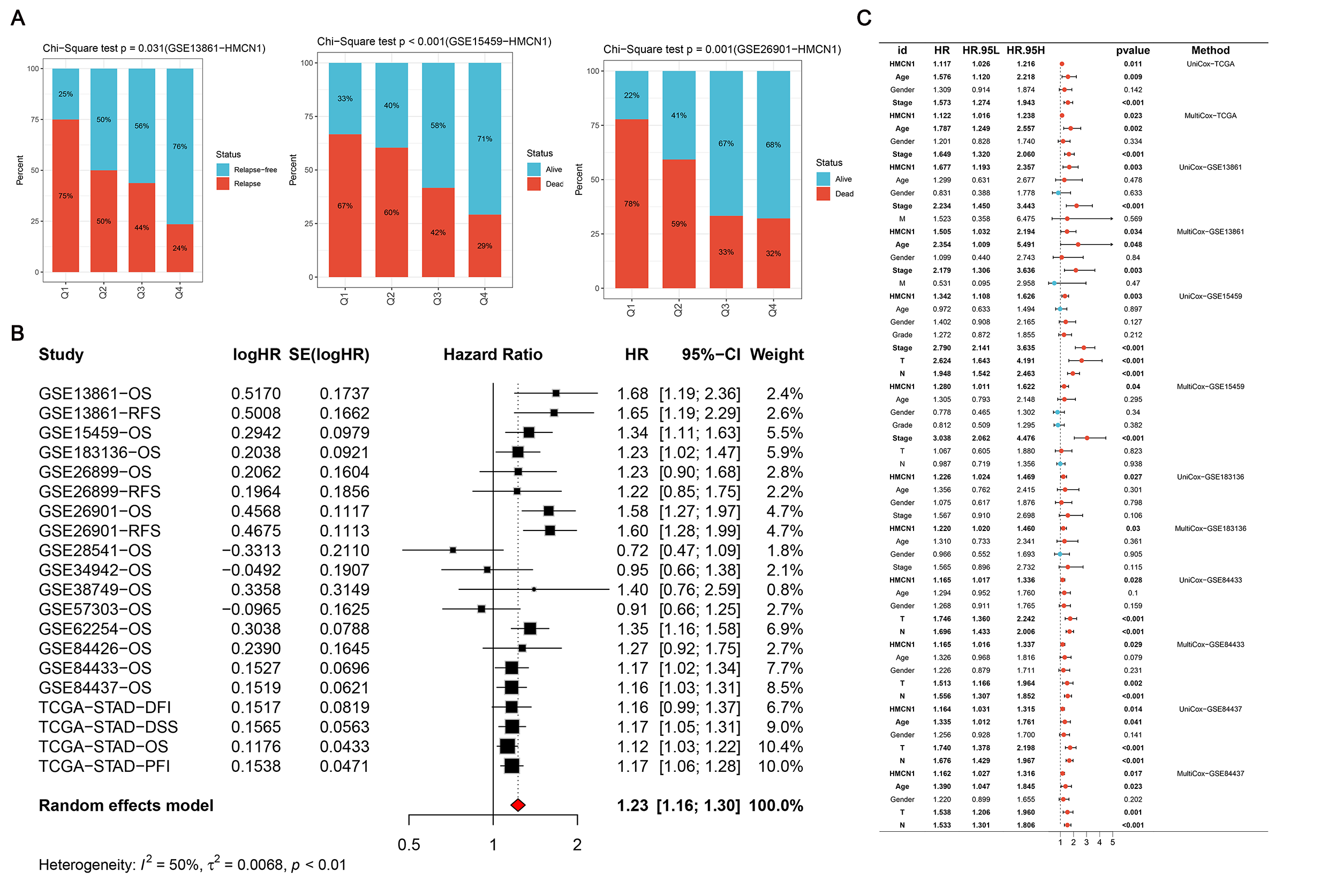

Supplement: Supplementary Figure 1 — (A, B) Bibliometric analysis of HMCN1 in oncology, showing the rising publication trend and the current research focus on prognosis, mutation, and therapy. (C, D) Single-cell RNA sequencing and spatial transcriptomics reveal the expression pattern of HMCN1. [file DataSheet1.zip › Supplementary files/Supplementary Figure 3.tif]

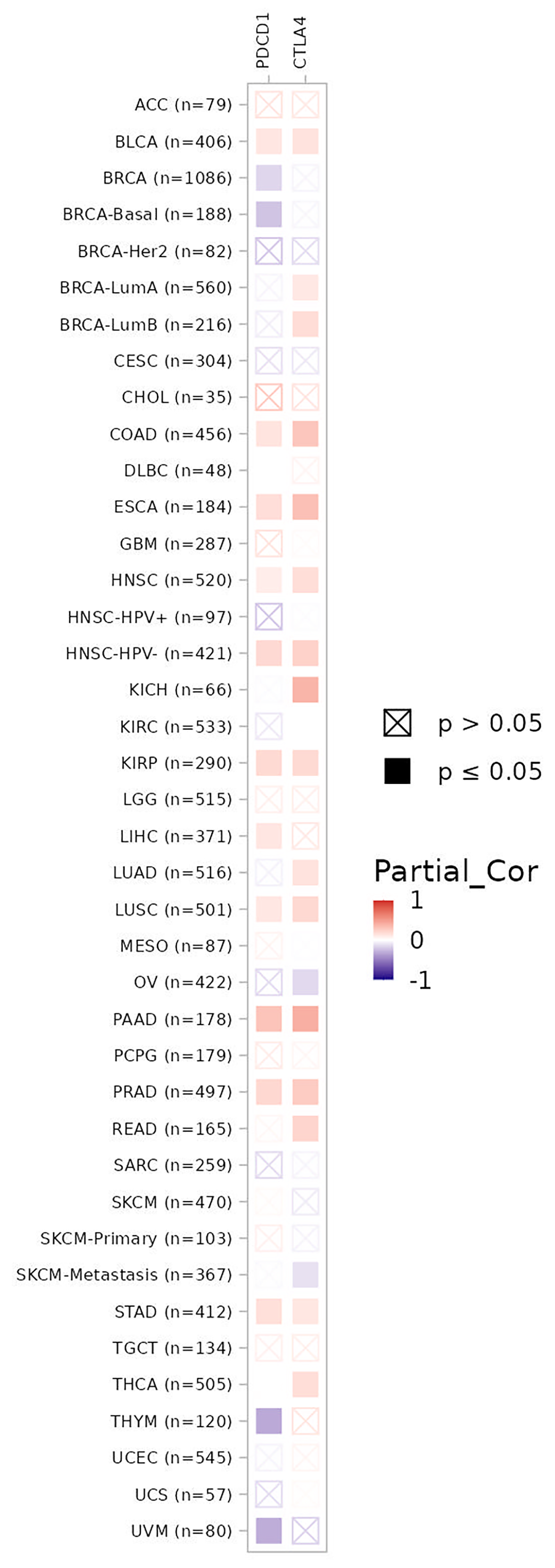

Supplement: Supplementary Figure 1 — (A, B) Bibliometric analysis of HMCN1 in oncology, showing the rising publication trend and the current research focus on prognosis, mutation, and therapy. (C, D) Single-cell RNA sequencing and spatial transcriptomics reveal the expression pattern of HMCN1. [file DataSheet1.zip › Supplementary files/Supplementary Figure 4.tif]
